# The maternal brain: Region‐specific patterns of brain aging are traceable decades after childbirth

**DOI:** 10.1002/hbm.25152

**Published:** 2020-08-07

**Authors:** Ann‐Marie G. de Lange, Claudia Barth, Tobias Kaufmann, Melis Anatürk, Sana Suri, Klaus P. Ebmeier, Lars T. Westlye

**Affiliations:** ^1^ Department of Psychiatry University of Oxford Oxford UK; ^2^ Department of Psychology University of Oslo Oslo Norway; ^3^ NORMENT, Institute of Clinical Medicine, University of Oslo, & Division of Mental Health and Addiction Oslo University Hospital Oslo Norway; ^4^ Wellcome Centre for Integrative Neuroimaging University of Oxford Oxford UK; ^5^ KG Jebsen Centre for Neurodevelopmental Disorders University of Oslo Oslo Norway

**Keywords:** brain‐age prediction, maternal brain aging, neuroimaging, parity

## Abstract

Pregnancy involves maternal brain adaptations, but little is known about how parity influences women's brain aging trajectories later in life. In this study, we replicated previous findings showing less apparent brain aging in women with a history of childbirths, and identified regional brain aging patterns linked to parity in 19,787 middle‐ and older‐aged women. Using novel applications of brain‐age prediction methods, we found that a higher number of previous childbirths were linked to less apparent brain aging in striatal and limbic regions. The strongest effect was found in the accumbens—a key region in the mesolimbic reward system, which plays an important role in maternal behavior. While only prospective longitudinal studies would be conclusive, our findings indicate that subcortical brain modulations during pregnancy and postpartum may be traceable decades after childbirth.

## Graphical abstract

In this study, we identified regional brain‐aging patterns linked to parity in 19,787 middle‐ and older‐aged women. A higher number of previous childbirths were linked to less apparent brain aging in striatal and limbic regions. The strongest effect was found in the accumbens—a key region in the mesolimbic reward system, which plays an important role in maternal behavior.

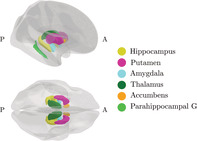

## 1 INTRODUCTION

Pregnancy involves a number of maternal brain adaptations (Barha & Galea, 2017; Boddy, Fortunato, Wilson Sayres, & Aktipis, 2015; Eid et al., 2019; Fox, Berzuini, Knapp, & Glynn, 2018; Hillerer, Jacobs, Fischer, & Aigner, 2014). In rodents, changes in volume, cell proliferation, and dendritic morphology (Hillerer et al., 2014; Kinsley et al., 2006), as well as altered neurogenesis in the hippocampus (Eid et al., 2019; Rolls, Schori, London, & Schwartz, 2008) are found across pregnancy and postpartum. In humans, reduction in total brain volume has been observed during pregnancy, reversing within 6 months of parturition (Oatridge et al., 2002). Reductions in striatal volumes, particularly putamen, have been reported shortly after delivery (Lisofsky et al., 2016), and pregnancy‐related reductions in gray matter volume have been found in regions subserving social cognition; the bilateral lateral prefrontal cortex, the anterior and posterior midline, and the bilateral temporal cortex (Hoekzema et al., 2017). Conversely, a recent study showed no evidence of decrease in gray matter volume following childbirth, but instead detected a pronounced gray matter *increase* in both cortical and subcortical regions (Luders et al., 2020). Prefrontal cortical thickness and subcortical volumes in limbic areas have been positively associated with postpartum months (Kim, Dufford, & Tribble, 2018), indicating that changes in brain structure may depend on region and time since delivery (Duarte‐Guterman, Leuner, & Galea, 2019; Hoekzema et al., 2017; Kim et al., 2010, 2018; Luders et al., 2020). For instance, from 2–4 weeks to 3–4 months postpartum, gray matter volume increases have been found in areas involved in maternal behaviors and motivation, such as the amygdala, substantia nigra, hypothalamus, and prefrontal cortex (Kim et al., 2010). Hence, the nature and magnitude of pregnancy‐related neural adaptations may be contingent on the specific time window during which they are measured.

While gray matter changes have been reported to endure up to 2 years postpregnancy (Hoekzema et al., 2017), most studies are limited to the postpartum period, and little is known about how previous pregnancies influence women's brain aging later in life. Evidence from animal studies shows that middle‐aged multiparous rats have stronger cellular response to estrogens in the hippocampus compared to virgin female rats (Barha & Galea, 2011), suggesting that neuroplastic potential across the adult lifespan may be influenced by previous pregnancies. Moreover, hippocampal neurogenesis has been shown to increase during middle age in primiparous rats and decrease in nulliparous rats over the same period (Eid et al., 2019). While longitudinal studies on parity and brain aging in humans are lacking, cumulative number of months pregnant has been associated with decreased risk for Alzheimer's disease (AD; Fox et al., 2018), and we recently documented less evident brain aging in parous relative to nulliparous women in >12,000 UK Biobank participants using an magnetic resonance imaging (MRI)‐derived biomarker of global brain aging (de Lange et al., 2019).

In the current study, we first aimed to replicate our previously reported findings described in de Lange et al. (2019), where less apparent brain aging was found in women with a history of childbirths. Brain‐age prediction was used to derive estimates of global brain aging, which was analyzed in relation to number of previous (live) childbirths in 8,880 newly added UK Biobank participants. Brain‐age prediction is commonly used to estimate an individual's age based on their brain characteristics (Cole & Franke, 2017), and individual variation in “brain age” estimates has been associated with a range of clinical and biological factors (Cole, 2020; Cole et al., 2017, 2018; Cole & Franke, 2017; Cole, Marioni, Harris, & Deary, 2019; de Lange, Anatürk, et al., 2020; de Lange, Barth, et al., 2020; Franke & Gaser, 2019; Kaufmann et al., 2019; Smith, Vidaurre, Alfaro‐Almagro, Nichols, & Miller, 2019). As compared to MRI‐derived measures such as cortical volume or thickness, brain‐age prediction adds a dimension by capturing deviations from normative aging trajectories identified by machine learning. While traditional brain‐age approaches summarize measures across regions to produce a single, global aging estimate—often with high prediction accuracy, models of distinct and regional aging patterns can provide more refined biomarkers that may capture additional biological detail (Eavani et al., 2018; Kaufmann et al., 2019; Smith et al., 2020). In this study, we utilized novel applications of brain‐age prediction methods based on cortical and subcortical volumes to identify regions of particular importance for maternal brain aging in 19,787 UK Biobank women.

## 2 METHODS AND MATERIALS

### 2.1 Sample characteristics

The sample was drawn from the UK Biobank (www.ukbiobank.ac.uk), and included 21,928 women. A total of 1,885 participants with known brain disorders were excluded based on ICD10 diagnoses (Chapters V and VI, field F; *mental and behavioral disorders*, including F00—F03 for AD and dementia, and F06.7 *Mild cognitive disorder*, which includes cognitive decline involving learning, memory, or concentration (World Health Organization, 1993) , and field G; *diseases of the nervous system*, including inflammatory and neurodegenerative diseases; except G55‐59; *Nerve*, *nerve root and plexus disorders*). An overview of the diagnoses is provided in the UK Biobank online resources (http://biobank.ndph.ox.ac.uk/showcase/field.cgi?id=41270), and the diagnostic criteria are listed in the ICD10 diagnostic manual (https://www.who.int/classifications/icd/icdonlineversions). A group of two hundred two participants was excluded based on MRI outliers (see Section 2.2; *MRI data acquisition and processing*) and 9 had missing data on number of previous childbirths, yielding a total of 19,787 participants that were included in the analyses. Sample demographics are provided in Table 1.

**TABLE 1:** Sample demographics

Age	
Mean ± *SD*	63.59 ± 7.38
Range (years)	45.13–82.27
Number of childbirths (live)	
Mean ± *SD*	1.72 ± 1.16
Range	0–9
*N* in each group:	
**0** = 4,297 | **1** = 2,459 | **2** = 8,770	
**3** = 3,334 | **4** = 729 | **5** = 142	
**6** = 43 | **7** = 7 | **8** = 5 | **9** = 1	
Age at first birth (*N* = 15,446)	
Mean ± *SD*	27.08 ± 5.01
Range	14–47
Years since last birth (*N* = 13,023)	
Mean ± *SD*	33.37 ± 9.32
Range	6.77–60.34
Menopausal status (*N* = 19,781)	
Yes	6,117
No	10,737
Not sure, had hysterectomy	1,912
Not sure, other reason	1,015
Ethnic background	
% White	97.06
% Black	0.69
% Mixed	0.54
% Asian	0.75
% Chinese	0.37
% Other	0.55
% Do not know	0.03
Education	
% University/college degree	44.71
% A levels or equivalent	14.09
% O levels/GCSE or equivalent	24.91
% NVQ or equivalent	3.17
% Professional qualification	5.65
% None of the above	5.91
Assessment location (imaging)	
Newcastle	5,139
Cheadle	11,906
Reading	2,742

Abbreviations: GCSE, General Certificate of Secondary Education; NVQ, National Vocational Qualification.

### 2.2 MRI data acquisition and processing

A detailed overview of the UK Biobank data acquisition and protocols is available in papers by Alfaro‐Almagro et al. (2018) and Miller et al. (2016). Raw T1‐weighted MRI data for all participants were processed using a harmonized analysis pipeline, including automated surface‐based morphometry and subcortical segmentation. Volumes of cortical and subcortical brain regions were extracted based on the Desikan‐Killiany atlas (Desikan et al., 2006) and automatic subcortical segmentation in FreeSurfer (version 5.3) (Fischl et al., 2002), yielding a set of 68 cortical features (34 per hemisphere) and 17 subcortical features (8 per hemisphere + the brain stem). The MRI data were residualized with respect to scanning site, data quality, and motion using Euler numbers (Rosen et al., 2018) extracted from FreeSurfer, intracranial volume (Voevodskaya et al., 2014), and ethnic background using linear models. To remove poor‐quality data likely due to subject motion, participants with Euler numbers of *SD* ± 4 were identified and excluded (*n* = 192). In addition, participants with *SD* ± 4 on the global MRI measures mean cortical or subcortical gray matter volume were excluded (*n* = 13 and *n* = 22, respectively), yielding a total of 19,796 participants with T1‐weighted MRI data. Only participants who had data on number of previous childbirths in addition to MRI were included, and the final sample used in all subsequent analyses (unless otherwise stated) counted 19,787 participants.

### 2.3 Brain‐age prediction

In line with recent brain‐age studies (de Lange et al., 2019; de Lange, Anatürk, et al., 2020; Kaufmann et al., 2019), the *XGBoost regressor model*, which is based on a decision‐tree ensemble algorithm (https://xgboost.readthedocs.io/en/latest/python), was used to estimate global and regional brain age based on the MRI data. XGboost includes advanced regularization to reduce overfitting (Chen & Guestrin, 2016), and uses a gradient boosting framework where the final model is based on a collection of individual models (https://github.com/dmlc/xgboost). Randomized search with 10 folds and 10 iterations was run to optimize parameters, using all imaging features as input. Scanned parameters ranges were set to *maximum depth*: (Barha & Galea, 2017; Fox et al., 2018; Hoekzema et al., 2017), *number of estimators*: [60, 220, 40], and *learning rate*: [0.1, 0.01, 0.05]. The optimized parameters *maximum depth* = 6, *number of estimators* = 140, and *learning rate* = 0.1 were used for all subsequent models.

### 2.4 Replication of previous findings

To replicate our findings described in de Lange et al. (2019), we trained a *global* brain‐age prediction model using the part of the current sample that overlapped with the previous study (*N* = 10,907) and applied it to the newly added participants (*N* = 8,880), yielding a global brain‐age estimate for each individual. Next, we calculated each participant's *brain‐age delta* by subtracting chronological age from estimated brain age. This measure provides an estimation of an individual's brain aging pattern relative to normative aging trajectories (Cole, 2020; Cole et al., 2018, 2019; Cole & Franke, 2017; de Lange et al., 2019; Franke & Gaser, 2019; Smith et al., 2019). For instance, if a 70‐year‐old individual exhibits a brain‐age delta of +5 years, their typical aging pattern resembles the brain structure of a 75 years old, that is, their estimated brain age is older than what is expected for their chronological age (Franke & Gaser, 2019). Finally, we tested the association between global brain aging and number of previous childbirths in the group of new participants, using a linear regression model with *brain‐age delta* as the dependent variable and *number of childbirths* as the independent variable. Chronological age was included as a covariate, adjusting for age‐bias in the brain‐age predictions as well as age‐dependence in number of childbirths (de Lange & Cole, 2020; Le et al., 2018). Note that the training set of 10,907 participants overlapping with the previous study showed a lower *N* relative to the original sample in (de Lange et al., 2019) due to variation in exclusion criteria (listed in Section 2.1; *Sample charecteristics*).

### 2.5 Regional brain aging patterns and associations with previous childbirths

The full sample (19,787) was utilized to investigate *regional* brain aging and associations with number of previous childbirths. Averages of the right and left hemisphere measures were first calculated for each MRI feature. Next, the MRI features were grouped together based on common covariance using hierarchical clustering on the Spearman rank‐order correlation in Scikit‐learn (version 0.22.2; https://scikit-learn.org/stable/modules/clustering). For each identified cluster of MRI features, separate prediction models were run with 10‐fold cross validation, providing *cluster‐specific* brain‐age delta estimates for each individual. To investigate model prediction accuracy, R^2^, root mean square error (RMSE), and mean absolute error (MAE) were calculated for each model, and correlation analyses were run for predicted versus chronological age. Associations with number of previous childbirths were investigated using separate regression analyses with cluster‐specific brain‐age delta estimates as the dependent variable, and *number of childbirths* as the independent variable. Chronological age was included as a covariate, and *p*‐values were adjusted for multiple comparisons using false discovery rate correction (Benjamini & Hochberg, 1995). To directly compare the associations, *Z* tests for correlated samples (Zimmerman, 2012) were run with

(1) $$Z=\left(\beta_{m1}-\beta_{m2}\right)/\sqrt{\sigma_{m1}^{2}+\sigma_{m2}^{2}-2\rho \sigma_{m1}\sigma_{m2}}.$$

where “m1” and “m2” represent models 1 and 2, the *β* terms represent the beta values from the regression fits, the *σ* terms represent their errors, and *ρ* represents the correlation between the two sets of associations.

To further identify specific regions associated with number of childbirths, the hierarchical clustering procedure was repeated on the MRI features within the cluster showing the strongest association with number of previous childbirths. For each identified subcluster, brain‐age prediction models were run to generate subcluster‐specific brain‐age estimates for each individual. Next, we tested the association between subcluster‐specific brain‐age estimates and number of childbirths. For the subcluster showing the strongest association, a linear regression was run to test the difference in subcluster‐specific brain aging between parous and nulliparous women. Subcluster‐specific brain age was entered as the dependent variable, and a binary variable for parity/nulliparity was used as the independent variable. Age was included as a covariate. To identify the unique contribution of each region contained in this cluster, separate brain‐age models were run with each MRI feature as input, and regression models were run with feature‐specific brain age as the dependent variable, and *number of childbirths* as the independent variable. Age was included as a covariate. All statistical analyses were conducted using Python 3.7.0.

## 3 RESULTS

### 3.1 Replication of previous findings

To replicate our findings described in de Lange et al. (2019), we applied the global brain‐age prediction model trained on the sub‐sample overlapping with the previous study (*N* = 10,907) to the newly added participants (*N* = 8,880). When applied to the test set, the modeled age prediction showed an accuracy of R^2^ = 0.34, RMSE = 6.00, and Pearson's *r* (predicted vs. chronological age) = 0.58, 95% confidence interval (CI) = [0.57, 0.59], *p* < .001. Corresponding to our previous results, an association was found between a higher number of previous childbirths and less apparent brain aging in the sample of newly added participants: *β* = −0.13, *SE* = 0.03, *t* = −4.07, *p* = 4.79 × 10^−5^.

**FIGURE 1:**
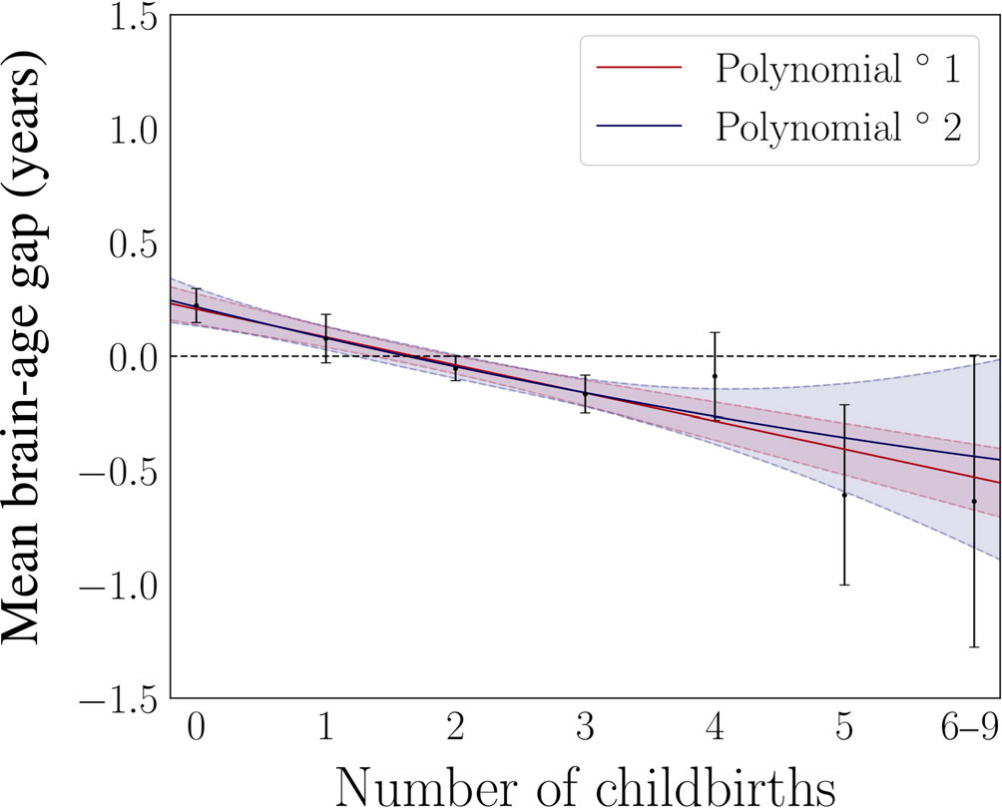
Results from first and second degree polynomial fits for number of childbirths and global brain aging in the newly added participants (*N* = 8,880). The black points indicate the mean brain age delta ±*SE* within groups of women based on number of childbirths (x‐axis). The red and blue lines represent the results of the fits, and the shaded areas indicate the 95% confidence intervals for each fit. The horizontal dashed line indicates 0 on the y‐axis. Number of participants in each group: 0 births = 2,065, 1 birth = 1,014, 2 births = 3,912, 3 births = 1,493, 4 births = 311, 5 births = 67, 6 births = 13, 7 births = 3, 8 births = 1, and 9 births = 1. The women with 6–9 children were merged into one gorup to obtain sufficient statistics for least square fits using the *SE* on the means as weights

To test for nonlinear relationships, polynomial fits were run for number of childbirths and brain age delta: one including intercept and a linear term (*β*) only, and one including intercept, linear, and quadratic terms (*γ*). For these analyses, the brain‐age delta estimations were first corrected for chronological age using linear regression (Le et al., 2018), and the residuals were used in the fits. A comparison of the two models showed that the inclusion of the quadratic term did not provide a better fit (*F* = 0.06, *p* = .804). The results from the fit including the linear term only showed a significant linear effect (*β* = −0.12 ± 0.03, *F* = 16.10, *p* = 6.05 × 10^−5^), while the results from the fit including both terms showed that only the linear term was significant (*β* = −0.14 ± 0.072, *γ* = 0.004 ± 0.02, *F* = 8.08, *p* = 3.11 × 10^−4^). The two fits are shown in Figure 1. As a cross check, the fits were rerun with orthogonal polynomials, showing corresponding results (*β* = −13.44 ± 3.35, *γ* = 0.83 ± 3.35, *F* = 8.08, *p* = 3.11 × 10^−4^).

### 3.2 Regional brain aging patterns and associations with previous childbirths

**FIGURE 2:**
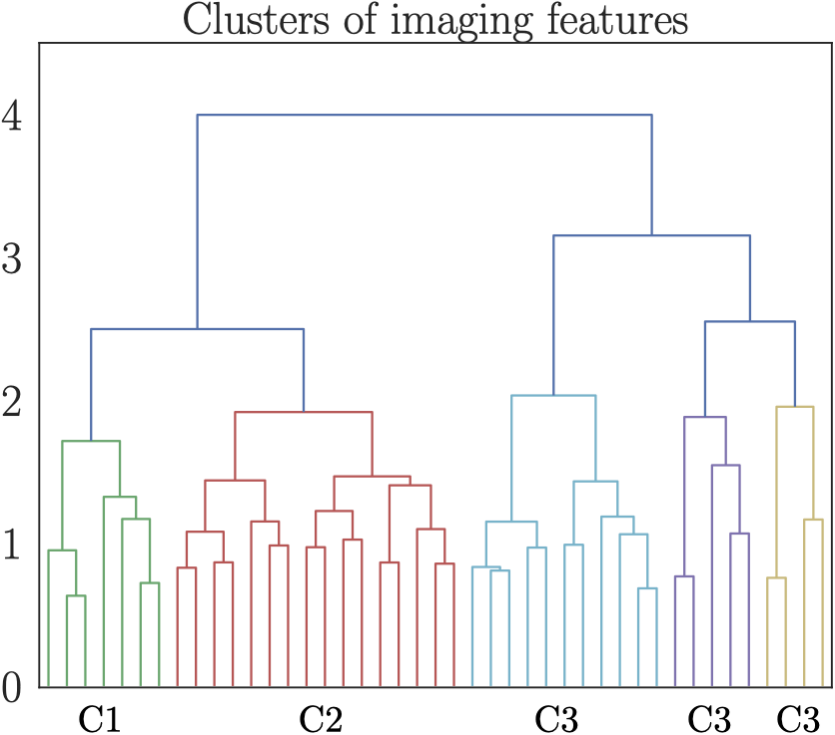
Dendrogram based on hierarchical clustering on the Spearman rank‐order correlations of all features. The colors represent clusters (C) of features that are grouped together based on common covariance. A list of the imaging features contained in each of the clusters is provided in Table 2. The y‐axis shows the degree of colinearity, with higher y‐values indicating less colinearity between clusters

Five clusters of MRI features were identified based on common covariance, as shown in Figure 2. The features contained in each cluster are listed in Table 2. Separate models were run to estimate brain age for each cluster using the brain‐age prediction procedure described in Section 2. The cluster‐specific model performances are shown in Table 3. To test whether the relative prediction accuracy of the models depended on number of features, the models were rerun using the four strongest contributing features from each model as input variables. The feature contributions were calculated using permutation feature importance, defining the decrease in model performance when a single feature value is randomly shuffled (Breiman, 2001). The results are shown in Table 4.

**TABLE 2:** List of imaging features contained in each of the clusters identified based on hierarchical clustering (Figure 2). All feature names represent regional volume

**Cluster 1**			
Cuneus	Isthmuscingulate	Lateraloccipital	Lingual
Pericalcarine	Precuneus	Superiorparietal	
**Cluster 2**			
Caudalanteriorcingulate	Lateralorbitofrontal	Medialorbitofrontal	Paracentral
Parsopercularis	Parsorbitalis	Parstriangularis	Postcentral
Posteriorcingulate	Precentral	Rostralmiddlefrontal	Superiorfrontal
Superiortemporal	Supramarginal	Transversetemporal	Insula
**Cluster 3**			
Banks of superior temporal sulcus	Fusiform	Inferiorparietal	Inferiortemporal
Middletemporal	Parahippocampal	Thalamus	Putamen
Hippocampus	Amygdala	Accumbens	
**Cluster 4**			
Caudalanteriorcingulate	Entorhinal	Rostralanteriorcingulate	Frontalpole
Temporalpole			
**Cluster 5**			
Brain‐stem	Cerebellum	Caudate	Pallidum

**TABLE 3:** The accuracy of the age prediction measured by Pearson's *r* (predicted vs. chronological age), R^2^, root mean square error (RMSE), and mean absolute error (MAE) for each of the cluster‐specific models. 95% confidence intervals are indicated in square brackets. RMSE and MAE are reported in years. *N*_feat_ represents the number of features contained in the cluster. *p*‐Values were <.001 for all models

Model	*N*_feat_	*r*	R^2^	RMSE	MAE
Cluster 1	7	0.32 [0.31, 0.33]	0.10 [0.10, 0.11]	7.00	5.79
Cluster 2	16	0.38 [0.36, 0.39]	0.14 [0.13, 0.15]	6.86	5.64
Cluster 3	11	0.48 [0.47, 0.49]	0.23 [0.23, 0.24]	6.48	5.29
Cluster 4	5	0.13 [0.11, 0.14]	0.02 [0.01, 0.02]	7.36	6.13
Cluster 5	4	0.21 [0.19, 0.22]	0.04 [0.04, 0.05]	7.25	6.04

**TABLE 4:** The accuracy of the age prediction when including the four strongest contributing features for each model. 95% confidence intervals are indicated in square brackets. Root mean square error (RMSE) and mean absolute error (MAE) are reported in years. *N*_feat_ represents the number of features included. *p*‐Values were <.001 for all models

Model	*N*_feat_	*r*	R^2^	RMSE	MAE
Cluster 1	4	0.32 [0.31, 0.33]	0.10 [0.10, 0.11]	7.01	5.80
Cluster 2	4	0.34 [0.33, 0.35]	0.11 [0.11, 0.12]	6.96	5.75
Cluster 3	4	0.46 [0.45, 0.47]	0.21 [0.21, 0.22]	6.55	5.36
Cluster 4	4	0.12 [0.11, 0.13]	0.02 [0.01, 0.02]	7.36	6.14
Cluster 5	4	0.21 [0.19, 0.22]	0.04 [0.04, 0.05]	7.25	6.04

**FIGURE 3:**
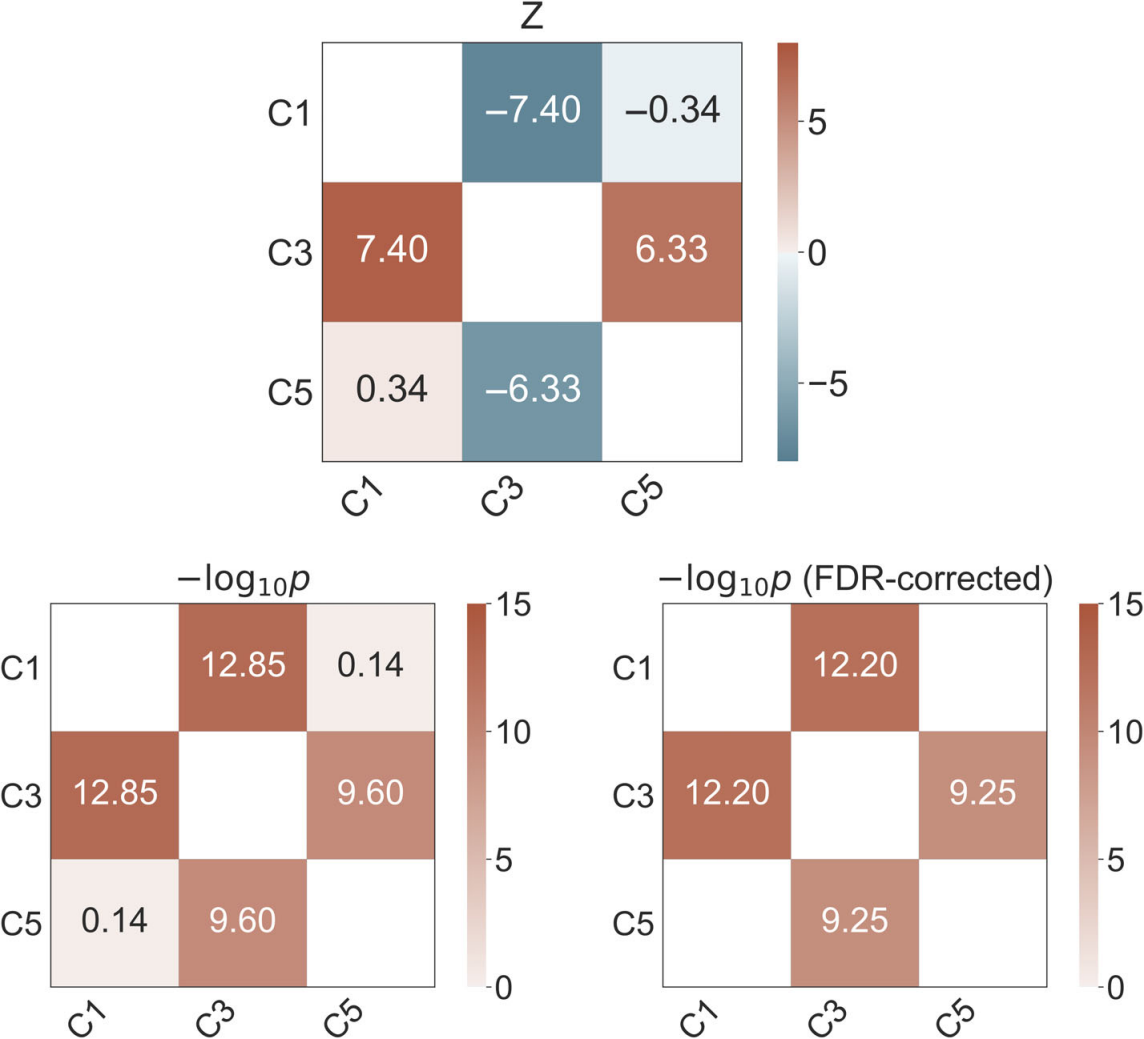
Statistical differences between cluster‐specific associations with number of childbirths. Top plot: Matrix showing pairwise differences between the significant cluster‐specific associations with number of childbirths, based on Z tests for correlated samples (Equation 1). Bottom left plot: Uncorrected −Log10 *p*‐values of the differences between the cluster‐specific associations. Bottom right plot: −Log10 *p*‐values corrected for multiple comparisons using false discovery rate correction (FDR), with only significant values (<.05) displayed. C, cluster

As shown in Table 5, brain aging estimates based on three clusters were each significantly associated with number of previous childbirths. To test whether the associations were statistically different from each other, pairwise *Z* tests for correlated samples (Equation 1; Section 2) were run on the cluster‐specific associations with number of childbirths. The results showed that Cluster 3 was more strongly related to number of previous childbirths relative to the other clusters, as shown in Figure 3.

**TABLE 5:** Relationships between number of previous childbirths and estimated brain aging for each cluster. Cluster‐specific *brain age delta* was entered as the dependent variable and number of (live) childbirths was entered as independent variable for each analysis. Chronological age was included for covariate purposes. *P*‐values are provided before and after FDR correction.

Number of childbirths vs. cluster‐specific brain‐age estimates					
Cluster	*β*	*SE*	*t*	*p*	*p _corr_*
1	−0.054	0.016	−3.282	.001	.002*
2	−0.010	0.018	−0.552	.581	.726
3	−0.133	0.021	−6.385	1.754 × 10^−10^	8.768 × 10^−10^*
4	−0.003	0.010	−0.278	.781	.781
5	−0.056	0.012	−4.620	3.868 × 10^−16^	9.670 × 10^−6^*

*significant relationships (< 0.05)

**FIGURE 4:**
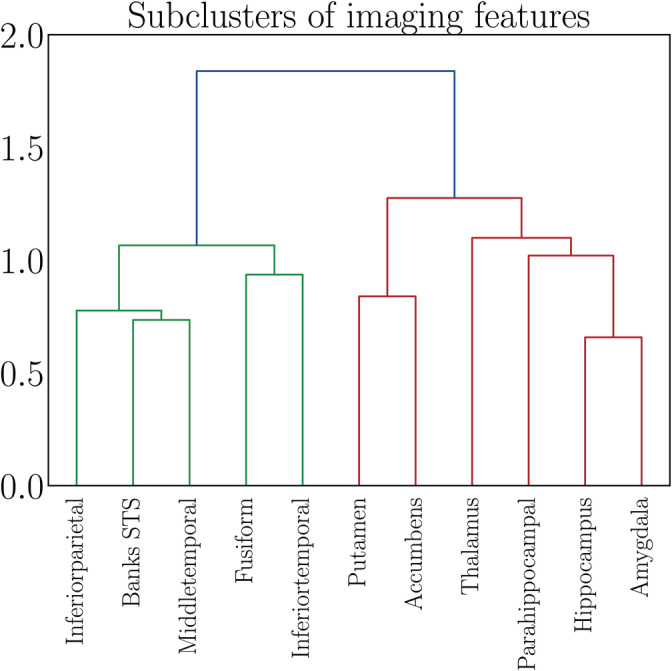
Dendrogram based on hierarchical clustering on the Spearman rank‐order correlations of the features contained in Cluster 3, which showed the strongest association with number of childbirths (see Figure 3). The colors represent clusters of features that are grouped together based on common covariance; subcluster 1 in green and subcluster 2 in red. The y‐axis shows the degree of colinearity, with higher y‐values indicating less colinearity between clusters. STS, superior temporal sulcus

**FIGURE 5:**
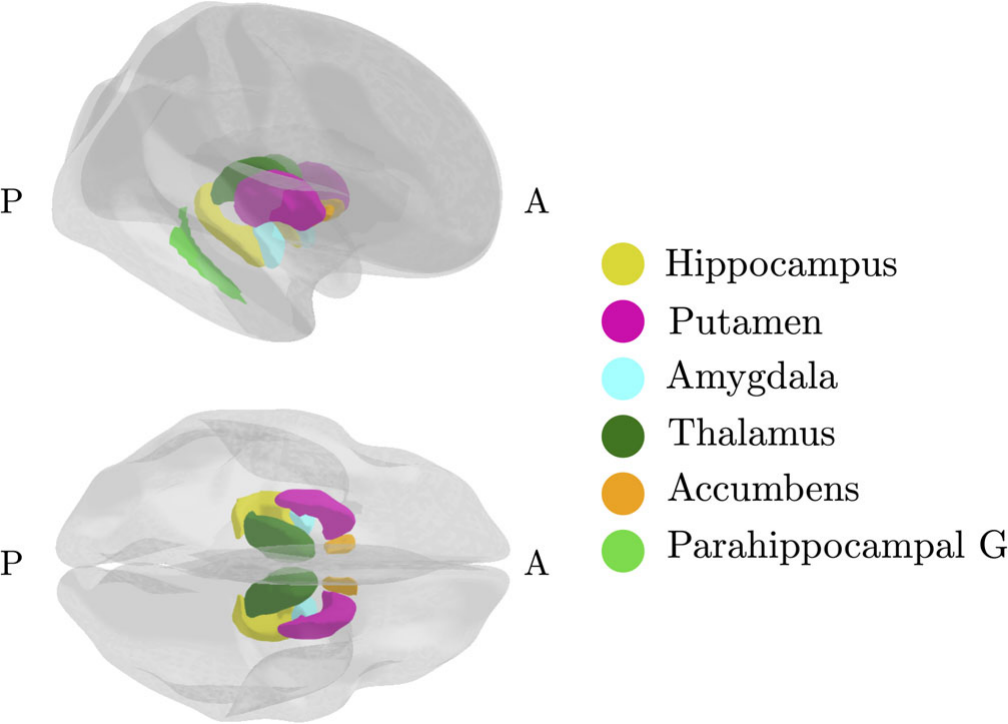
Regions in subcluster 2—the cluster that showed the strongest association with number of previous childbirths. A, anterior; G, gyrus; P, posterior. Figure created using the *ggseg* plotting tool for brain atlases in R (Mowinckel & Vidal‐Piñeiro, 2019)

To investigate further specificity, the clustering procedure was repeated on the features in Cluster 3—the cluster showing the strongest association with number of childbirths. Two subclusters were identified based on the covariance of the features: subcluster 1 included volume in the regions inferiorparietal, middletemporal, inferiortemporal, fusiform, and banks of superior temporal sulcus, while subcluster 2 included putamen, accumbens, thalamus, hippocampus, amygdala, and parahippocampal gyrus, as shown in Figure 4. Separate models were run to generate brain‐age predictions for each of the two subclusters. The subcluster‐specific model performances are shown in Table 6, and their associations with number of previous childbirths are shown in Table 7. The results showed that subcluster 2 was more strongly related to number of previous childbirths, as shown in Table 8. The regions in subcluster 2 are shown in Figure 5.

**TABLE 6:** The accuracy of the age prediction measured by Pearson's *r* (predicted vs. chronological age), R^2^, root mean square error (RMSE), and mean absolute error (MAE) for each of the subcluster‐specific models. 95% confidence intervals are indicated in square brackets. RMSE and MAE are reported in years. *N*_feat_ represents the number of features contained in the cluster. *p*‐Values were <.001 for both models

Model	*N*_feat_	*r*	R^2^	RMSE	MAE
Subcluster 1	5	0.33 [0.31, 0.34]	0.11 [0.10, 0.12]	6.99	5.79
Subcluster 2	6	0.47 [0.45, 0.48]	0.22 [0.20, 0.23]	6.54	5.35

**TABLE 8:** Difference between the subcluster‐specific associations with number of childbirths, calculated using Equation (1)

Comparison	*Z*	*p*	*p*_corr_
Subcluster 1 vs. subcluster 2	−5.32	1.01 × 10^−7^	2.03 × 10^−7^

As subcluster 2 showed the strongest association with number of previous childbirths, a linear regression was performed to test for differences in subcluster 2‐specific brain aging between parous (*N* = 15,490) and nulliparous (*N* = 4,297) women. The results showed less apparent brain aging in parous relative to nulliparous women (*β* = −0.38, *SE* = 0.06, *t* = −6.81, *p* = 1.01 × 10^−11^), with an effect size of *d* = 0.12 ± 0.02 (*SE*).

**TABLE 7:** Relationships between number of previous childbirths and estimated brain aging for each subcluster. Subcluster‐specific brain age delta was entered as the dependent variable and number of childbirths was entered as independent variable for each analysis. Chronological age was included for covariate purposes. *P*‐values are provided before and after FDR correction

Number of childbirths vs. subcluster‐specific brain‐age estimates					
Subcluster	*β*	*SE*	*t*	*p*	*p _corr_*
1	−0.071	0.016	−4.321	1.563 × 10^−5^	1.563 × 10^−5^
2	−0.124	0.020	−6.169	7.024 × 10^−10^	1.405 × 10^−9^

#### 3.2.1 Adjusting for potential confounding factors

To control for potential confounding factors, the analyses of number of previous childbirths versus subcluster 2 brain‐age estimates were rerun including assessment location, education, body mass index (BMI), diabetic status, hypertension, smoking and alcohol intake, menopausal status (“yes,” “no,” “not sure, had hysterectomy,” and “not sure, other reason”), and oral contraceptive (OC) and hormonal replacement therapy (HRT) status (previous or current user vs. never used) as covariates. A total of, 16,512 women had data on all variables and were included in the analyses. The results showed an association of *β* = −0.12, *SE* = 0.02, *t* = −5.36, *p* = 8.27 × 10^−8^ between number of childbirths and subcluster 2, indicating that the covariates could not fully explain the association. To test whether gestational diabetes influenced brain aging among parous women, we analyzed available data on women who experienced gestational diabetes (*N* = 61) and women who did not (*N* = 234). A linear regression showed no association between gestational diabetes and subcluster 2 brain‐age estimates (*β* = −0.17, *SE* = 0.53, *t* = −0.33, *p* = .74, 95% CI = [−1.22, 0.87]. To further control for potential effects of pregnancy complications, we excluded women with any of the conditions listed in the ICD10 Chapter XV, *Pregnancy*, *childbirth and the puerperium* (*N* = 339; http://biobank.ndph.ox.ac.uk/showcase/field.cgi?id=41270), and repeated the main analysis including all covariates. The results showed an association of *β* = −0.12, *SE* = 0.02, *t* = −5.42, *p* = 5.92 × 10^−8^ between number of childbirths and subcluster 2.

As fetal sex has been shown to influence cognitive function during pregnancy, we extracted the available data from UK Biobank including sex of the firstborn child for 1,408 women to test for effects of fetal sex on brain aging. In this subsample, a linear regression showed no association between fetal sex and subcluster 2 brain‐age estimates (*β* = 0.12, *SE* = 0.16, *t* = 0.71, *p* = .48, 95% CI = [−0.20, 0.43]; women with male firstborn: 630, women with female firstborn: 778). Number of previous childbirths and age at first birth correlated *r* = −0.294, *p* = 6.90 × 10^−296^ (corrected for age). To test for an association with brain aging, an analysis was run with Subcluster 2 brain age as the dependent variable and *age at first birth* as the independent variable, including all covariates (age, assessment location, education, BMI, diabetic status, hypertension, smoking and alcohol intake, menopausal status, OC and HRT use). No association was found (*β* = 0.010, *SE* = 0.01, *t* = 1.64, *p* = .102, *N* = 12,937).

**TABLE 9:** Region‐specific age prediction accuracy (correlation between predicted and chronological age; *r* Age) and association with number of childbirths (*β* CB, standard error (SE), t, *p*, and *pcorr*) for each of the region‐specific brain age delta estimates. Chronological age was included in the analyses for covariate purposes. 95 % confidence intervals are indicated in square brackets. *P*‐values are reported before and after FDR correction

Subcluster 2 region	*r*_Age_	*β*_CB_	*SE*	*t*	*p*	*p*_corr_
Parahippo campal	0.24 [0.23, 0.25]	−0.020	0.012	−1.650	.099	.099
Thalamus	0.35 [0.34, 0.36]	−0.048	0.016	−2.991	.003	.003
Putamen	0.24 [0.22, 0.25]	−0.053	0.012	−4.401	1.08 × 10^−5^	2.253 × 10^−5^
Hippocampus	0.33 [0.32, 0.34]	−0.061	0.016	−3.923	8.77 × 10^−5^	1.315 × 10^−4^
Amygdala	0.29 [0.27, 0.30]	−0.061	0.014	−4.393	1.13 × 10^−5^	2.252 × 10^−5^
Accumbens	0.31 [0.30, 0.32]	−0.101	0.015	−6.812	9.90 × 10^−12^	5.937 × 10^−11^

**TABLE 10:** Difference in log‐likelihood (ΔLL) between regression analyses against number of children. The difference is calculated between models where all cluster features are included and models where single features are left out one at the time. *P*‐values are reported before and after FDR correction

Left‐out feature	Δ*LL*	*Z*	*p*	*p*_corr_
Parahippocampal	0.051	0.322	.758	.758
Thalamus	0.753	1.227	.376	.563
Putamen	1.819	1.907	.129	.388
Hippocampus	0.352	0.839	.561	.673
Amygdala	0.813	1.275	.354	.563
Accumbens	10.568	4.597	2.05 × 10^−5^	1.232 × 10^−4^

#### 3.2.2 Single‐region associations

To investigate the unique contributions of each region in subcluster 2 to the association with previous childbirths, separate brain‐age prediction models were run with each feature as input, yielding 11 region‐specific brain‐age estimates. Table 9 shows the correlation between predicted and chronological age for each region‐specific model, and their associations with number of childbirths. As the regions within the cluster were correlated (see Figure 4), we tested for unique contributions by first running a multiple regression analysis with all region‐specific brain‐age estimates as independent variables and *number of childbirths* as the dependent variable, before eliminating the regions one at a time to compare the log‐likelihood of the full and reduced models. The significance of model differences was calculated using Wilk's theorem (Wilks, 1938) as $\sqrt{2\left(∆\mathrm{LL}\right)}$, where Δ*LL* = *LL*_1_ − *LL*_2_; the difference in log‐likelihood between the reduced model (*LL*_1_) and the full model (*LL*_2_). The results showed that only the accumbens contributed uniquely to the association with number of previous childbirths, as shown in Table 10. The association when excluding accumbens from subcluster 2 was *β* = −0.098, *SE* = 0.020, *t* = −5.001, *p* = 5.766 × 10^−07^, indicating that the association was not solely driven by this region.

**TABLE 11:** Relationships between number of previous childbirths and volume for each region in subcluster 2. *P*‐values are provided before and after FDR correction

Number of childbirths vs. regional volume					
Region	*β*	*SE*	*t*	*p*	*p _corr_*
Parahippocampal	1.730	1.647	1.051	.293	.293
Thalamus	7.364	3.627	2.030	.042	.051
Putamen	9.369	3.215	2.914	.004	.005
Hippocampus	7.359	2.295	3.207	.001	.003
Amygdala	4.946	1.094	4.522	6.165 × 10^−6^	1.849 × 10^−5^
Accumbens	3.311	0.554	5.979	2.291 × 10^−9^	1.374 × 10^−8^

As a cross check, we investigated associations between previous childbirths and regional volumes in subcluster 2. Separate analyses were run with the volume measure for each region as dependent variables and number of previous childbirths as the independent variable, including age, assessment location, education, BMI, diabetic status, hypertension, smoking and alcohol intake, menopausal status, and OC and HRT status as covariates. In all, 16,516 women had data on all variables and were included in the analysis. The associations between number of previous childbirths and regional volume corresponded to the associations with brain‐age estimates, as shown in Table 11.

## 4 DISCUSSION

The results showed that a higher number of previous childbirths were associated with less apparent brain aging in striatal and limbic regions, including the accumbens, putamen, thalamus, hippocampus, and amygdala. The most prominent effect was seen in the accumbens, which is part of the ventral striatum and a key region in the mesolimbic system involved in reward processing and reinforcement learning (Haber & Knutson, 2010). The mesolimbic system plays a pivotal role in the rapid emergence of adequate maternal behavior directly after birth due to its role in motivation, reward, and the hedonic value of stimuli (Brunton & Russell, 2008; Numan & Woodside, 2010). In rodents, this circuit is activated by pup‐related cues that strongly motivate and reinforce maternal care, such as odor (Fleming, Cheung, Myhal, & Kessler, 1989), ultrasonic vocalization (Robinson, Zitzman, & Williams, 2011), and suckling (Ferris et al., 2005). Low levels of maternal care have been associated with reduced dopamine release within the nucleus accumbens in response to pup cues (Champagne et al., 2004), and in humans, motherhood has been linked to anatomical changes in the ventral striatum, with volume reductions promoting responsivity to offspring cues (Hoekzema et al., 2020). Together with the ventral striatum, regions including the thalamus, parietal cortex, and brainstem also serve important functions for processing pup‐related somatosensory information (Kim et al., 2010), and some evidence suggests that structural reorganization occurs in the thalamus, parietal lobe, and somatosensory cortex as a result of physical interactions with pups during nursing (Kinsley et al., 2008; Xerri, Stern, & Merzenich, 1994). A recent study by Luders et al. (2020) found an increase in regional volumes including the thalamus in women postpartum, corroborating functional MRI studies showing maternal thalamic activation in response to their offspring (Paul et al., 2019; Rocchetti et al., 2014). During mother–infant interaction, brain activation has also been shown to increase in the striatum (including putamen and accumbens), amygdala, substantia nigra, insula, inferior frontal gyrus, and temporal gyrus (Rocchetti et al., 2014). To summarize, the brain regions identified in the current study largely overlap with neural circuitry underpinning maternal behavior, indicating that brain modulations during pregnancy and postpartum may be traceable decades after childbirth.

In addition to the regions overlapping with the maternal circuit, we found a link between hippocampal brain aging and previous childbirths. This association concurs with animal studies showing enhanced hippocampal neurogenesis in middle age in parous relative to nulliparous rats (Eid et al., 2019), and fewer hippocampal deposits of amyloid precursor protein in multiparous relative to primiparous and virgin animals (Love et al., 2005). Contrary to the findings in middle‐aged animals, *reduced* hippocampal neurogenesis has been reported during the postpartum period, coinciding with enhanced memory performance in primiparous compared to nulliparous rodents (Kinsley & Lambert, 2008). In combination with the evidence of both increased and decreased regional volume in humans postpartum (Hoekzema et al., 2017; Kim et al., 2018; Luders et al., 2020), these findings emphasize that pregnancy‐related brain changes may be highly dynamic.

Pregnancy represents a period of enhanced neuroplasticity of which several underlying mechanisms could confer long‐lasting effects on the brain. Fluctuations in hormones including estradiol, progesterone, prolactin, oxytocin, and cortisol are known to influence brain plasticity (Barha & Galea, 2010; Galea, Leuner, & Slattery, 2014; Simerly, 2002), and levels of estradiol—a potent regulator of neuroplasticity (Barha & Galea, 2010)—rise up to 300‐fold during pregnancy (Schock et al., 2016) and fall 100–1,000 fold postnatally (Nott, Franklin, Armitage, & Gelder, 1976). Hormonal modulations are closely linked to pregnancy‐related immune adaptations such as the proliferation of Treg cells (Kieffer, Faas, Scherjon, & Prins, 2017), which promotes an anti‐inflammatory immune environment and contribute to the observed improvement in symptoms of autoimmune disease during pregnancy (Natri, Garcia, Buetow, Trumble, & Wilson, 2019; Whitacre et al., 1999). In contrast, the transition to menopause marks a period of decline in ovarian hormone levels and can foster a pro‐inflammatory phenotype involving increased risk for autoimmune activity and neuronal injury. Beneficial immune adaptations in pregnancy could potentially have long‐lasting effects, improving the response to menopause‐related inflammation, and subsequently leading to more favorable brain aging trajectories in multiparous women (Barth & de Lange, 2020; Fox et al., 2018; Mishra & Brinton, 2018). Another mechanism through which pregnancy may have long‐lasting effects on maternal physiology is fetal microchimerism—the presence of fetal cells in the maternal body (Boddy et al., 2015). In mice, fetal cells have been found in several brain regions including the hippocampus, where they mature into neurons and integrate into the existing circuitry (Zeng et al., 2010). Further evidence for beneficial effects of childbirths on the aging brain stems from studies showing that telomeres are significantly elongated in parous relative to nulliparous women (Barha et al., 2016), indicating that parity may slow the pace of cellular aging. However, parity has also been linked to AD‐like brain pathology including neurofibrillary tangle and neuritic plaque (Beeri et al., 2009; Chan et al., 2012), as well as increased risk of AD (Beeri et al., 2009; Colucci et al., 2006), particularly in grand‐parous women (>5 childbirths; Jang et al., 2018). Although our previous study showed some evidence of a moderate nonlinear trend between number of childbirths and global brain aging (de Lange et al., 2019), this effect was not replicated in the current study. More research is needed to determine whether positive effects of pregnancies are less pronounced in grand‐parous women, as our findings could be biased by the low number of women with five or more childbirths, as well as confounding factors such as socioeconomic status or stress levels (Zeng et al., 2016). Furthermore, as individuals with dementia, AD, and cognitive impairment were excluded from the study, the current findings do not provide information about any potential links between grand parity and risk for AD or other neurodegenerative diseases.

In conclusion, the current study replicates preceding findings showing less apparent brain aging in multiparous women (de Lange et al., 2019), and highlights brain regions that may be particularly influenced by previous childbirths. While prospective longitudinal studies are needed to fully understand any enduring effects of pregnancy, our novel use of regional brain‐age prediction—which captures deviations from normative aging—demonstrates that parity relates to region‐specific brain aging patterns evident decades after a woman's last childbirth.

## Data Availability

The data that support the findings of this study are available through the UK Biobank data access procedures (https://www.ukbiobank.ac.uk/researchers).
